# Physical activity on prescription in Swedish primary care: a survey on use, views, and implementation determinants amongst general practitioners

**DOI:** 10.1080/02813432.2023.2288126

**Published:** 2024-02-07

**Authors:** Elina Brorsson Lundqvist, Marcus Praetorius Björk, Susanne Bernhardsson

**Affiliations:** a University of Gothenburg, Sahlgrenska Academy, Gothenburg, Sweden; b Region Västra Götaland, Research, Education, Development, and Innovation Primary health care, Gothenburg, Sweden; c School of Public Health and Community Medicine, University of Gothenburg, Sahlgrenska Academy, Gothenburg, Sweden; d Institute of Neuroscience and Physiology, Department of Health and Rehabilitation, Unit of Physiotherapy, University of Gothenburg, Sahlgrenska Academy, Gothenburg, Sweden

**Keywords:** Physical activity on prescription, implementation, general practitioners, primary care, Normalization Process Theory

## Abstract

**Introduction:**

Swedish Physical Activity on Prescription (PAP) has been shown to increase physical activity levels, which is known to lead to positive health effects. PAP is being implemented in Swedish healthcare to various extents. However, there is a lack of knowledge about how Swedish general practitioners (GPs) work with PAP and what hinders and facilitates wider implementation.

**Aims:**

This study aimed to survey GPs’ use and views of PAP, identify barriers and facilitators for implementing PAP, and explore associations to gender, practice location, and experience.

**Methods:**

The study was framed by the Normalization Process Theory. A survey was sent to 463 GPs at 69 different healthcare centres in Region Västra Götaland. Data were analysed using multiple logistic and linear regressions.

**Results:**

A total of 143 GPs completed the survey (response rate 31%). Views on PAP were generally positive amongst respondents, but only 27% reported using PAP regularly. The most prominent reported barriers were insufficient training and resources. Positive views and willingness to collaborate in using PAP were identified as facilitators. Responding GPs in Gothenburg used PAP more often (OR 6.4; 95% CI 2.7–14.8) and were significantly more positive to the method than GPs in other areas of the region. GPs with more than 10 years of practice used PAP more often (OR 2.5; 95% CI 1.1–6.0) than less experienced GPs. Few of the investigated variables were associated with gender.

**Conclusions:**

The positive views amongst responding GPs are helpful, but more education, training and resources are needed for successful implementation of PAP in Swedish primary health care.

## KEY POINTS

- Implementation of physical activity on prescription (PAP) in Swedish healthcare has been ongoing for many years, but is implemented to various extents across and within regions.
- Views on PAP are generally positive, but use is not widespread amongst general practitioners in Region Västra Götaland who responded to the survey.
- The method is more frequently used in Gothenburg, where the most extensive implementation work has been done, and amongst more experienced general practitioners.
- Education and training in using PAP need to increase for successful implementation.

## Introduction

Physical inactivity and a sedentary lifestyle are today a substantial risk factor for both morbidity and mortality, accounting for over 7% of all-cause deaths globally [1] and up to 8% of all non-communicable diseases. Sedentary behaviours have also been associated with increased risk of all-cause mortality, cardiovascular disease mortality, cancer mortality, and type 2 diabetes [2]. According to self-reported data, 34% of the Swedish population do not reach recommended PA levels [3].

The benefits of PA on both physical and mental health are well established. A higher PA level combined with lower sedentary time decrease the risk for premature mortality [4] and can contribute to considerable health and economic benefits in society [5]. Physical activity reduces the risk for e.g. cardiovascular diseases, type 2 diabetes, and some types of cancer [6, 7]. It also improves mental health, cognitive abilities, and sleep and can decrease the risk for overweight, hypertension, high cholesterol, and hyperglycaemia [2].

The WHO has a goal to reduce physical inactivity by 15% from 2016 to 2030 [8]. Primary healthcare is a suitable arena to work towards this goal. In Sweden, primary healthcare reaches a substantial part of the population in need of lifestyle counselling, with more than 36 million appointments with a healthcare professional in 2021 [9], and is thereby well positioned for providing health promotion interventions. Ways of promoting PA in primary healthcare vary from brief advice and counselling in daily practice to more advanced methods, including PA prescriptions and use of behavioural change and cognitive approaches [10]. Recent systematic reviews have shown mixed results regarding the effect of PA interventions on PA levels [11, 12], although interventions that include follow-up and behavioural change strategies seem to yield a better outcome [11–13] and increase the chance to meet the PA guidelines of the WHO [13].

Counselling about PA in clinical practice has been shown to increase PA by 12%–50% [14]. If written prescription, diaries, pedometers, and informational brochures are used, PA levels can increase by another 15%–50%. Physical activity on prescription (PAP) is a specific counselling form, developed in Sweden in the early 2000s as a tool for healthcare practitioners to support patients in motivating and increasing PA. The Swedish PAP method comprises three core components: *a person-centred dialogue, an individually tailored PA prescription,* and *structured, individualised follow-up* [15]. In the *person-centred dialogue* the patient’s previous experience and attitude to PA are explored and their starting point and goal concerning PA are identified. The *individually tailored prescription* consists of a PA recommendation that is individually tailored regarding type, duration, frequency, and intensity. In the *structured follow-up,* the patient’s motivation, self-efficacy, and compliance to the prescription are evaluated, and if needed the agreed PA and prescription can be modified. In addition, two supplementary components for methodological support are *the ‘FYSS’^1^ knowledge support* and *collaboration with activity organisers* [15].

The method can be used both for prevention and treatment of many various diseases [15], and its use is recommended in national guidelines for prevention and treatment of unhealthy lifestyles [16]. Physical activity on prescription schemes have been launched in several countries in different variations and under different names [17], but Swedish PAP is considered *best practice* by both WHO and EU [18, 19]. Swedish PAP has been shown to increase PA levels and lead to positive health effects among physically inactive patients [14, 20, 21]. Sustained effects of PAP have been shown on PA level, metabolic health, and health-related quality of life five years after a PAP intervention in Swedish primary care [22]. Patients with metabolic risk factors have described increased motivation for PA and that they experienced many health benefits after having received a tailored prescription for PA [23].

In Sweden all licensed healthcare professionals, including general practitioners (GPs), with sufficient knowledge on the subject can prescribe PAP [24]. General practitioners are in a particularly good position to work with PAP as they see most patient groups, of all ages, that are at risk for e.g. metabolic or cardiovascular disease, diabetes, obesity and other lifestyle-related issues and are a credible source of lifestyle advice [25, 26]. Two thirds of all visits in primary healthcare in 2021 were to a GP [9]. A majority of patients presenting in general practice are physically inactive, and many desire help from their GP to increase their physical activity [27].

Implementing and incorporating new methods into clinical practice is a process that takes time, especially when the new method is complex [28]. Using an implementation theory when studying the implementation of a method can help us better understand why an implementation succeeds or fails, which can facilitate further implementation work [29]. Particularly relevant to help understand the implementation of complex interventions in healthcare settings, is the Normalisation Process Theory (NPT) [30]. Normalisation is the process in which a new method is routinely embedded and becomes a natural part of clinical everyday practice [30]. The NPT was constructed to recognise what change needs to be done in both the individual and collective work to improve the implementation process. The theory consists of four core constructs, or domains (Table 1), representing different types of work that is performed during implementation [31].

**Table 1.** The four domains in the Normalisation Process Theory explained [31].

Domain	Explanation
*Coherence*	Understanding the benefits and purpose with an intervention, such as PAP. Also referred to as ‘sense-making work’.
*Cognitive participation*	The relational work that builds and sustains people’s development together within an area, also called ‘community of practice’.
*Collective action*	The operational work that is needed to implement an intervention, such as PAP.
*Reflexive monitoring*	The assessment that is done to understand how an intervention, such as PAP, affect ourselves and others around us.

Two qualitative studies on the experience of PAP amongst Swedish healthcare professionals showed similar results about what factors are required to improve and facilitate implementation of PAP in Swedish healthcare [32, 33]. They both emphasised the need for increased knowledge about the method, training, and clearer local routines. Barriers described were lack of time to use PAP in daily clinical practice and that the method was sometimes met by distrust. Local and central support structures, such as PAP coordinators, and collaboration with other professions, particularly physiotherapists, were considered essential to facilitate the implementation of PAP. To our knowledge, no study has investigated use of PAP employing an implementation theory. Knowledge about hindering and facilitating factors is important to understand how use of PAP can be increased. This study aimed to survey GPs’ use and views of PAP and identify barriers and facilitators for implementing PAP amongst GPs in Region Västra Götaland. This knowledge about implementation determinants is important as PAP is implemented sparsely and unevenly, despite being introduced into Swedish healthcare more than 20 years ago [34]. The study also aimed to explore associations between use and views of PAP with the GPs’ gender, practice location, and years of practice.

## Material and methods

### Study design

This study is a cross-sectional quantitative survey of GPs employed at primary healthcare centres (HCCs) in Region Västra Götaland, conducted in March-April 2022.

### Study setting

Region Västra Götaland is Sweden’s second largest regional authority (formerly called county councils) and provide healthcare services to 1.7 million inhabitants in western Sweden. Here and in other regions in Sweden, there is ongoing work to implement PAP, but the offered support to do so varies considerably [34]. In Region Västra Götaland, the Centre for Physical Activity Gothenburg was established in 2018, offering education, training, specialised PAP clinics, and research to support the implementation of PAP in the city of Gothenburg [35]. These services have primarily targeted primary health care in Gothenburg; hence, this is where the most extensive implementation work in the region has been done.

### Study population

**Figure 1.**
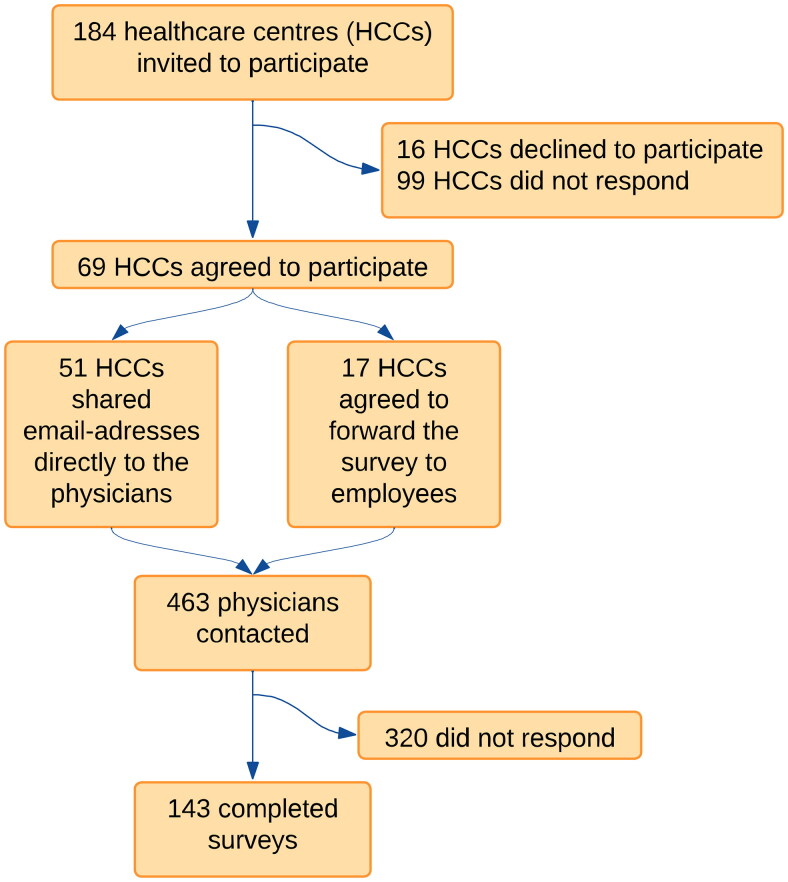
Participant flow through the study.

With permission from area managers, 184 HCCs (80 public and all 104 private HCCs in the region) were invited to participate in the study. Sixty-nine HCCs (36 private and 33 public) accepted the invitation. The survey was distributed to 463 GPs, resident GPs and other licensed physicians employed at the HCC, of whom 143 GPs responded to the survey (Figure 1).

### Data collection

The survey was based on the Normalisation Process Theory Measure (NoMAD), a validated questionnaire for studying implementation of new interventions developed from the NPT [36]. The NoMAD instrument consists of 23 items, of which three are general items about the intervention and 20 are detailed items about the intervention. The detailed items are developed from the domains in the theory and measures coherence, cognitive participation, collective action, and reflexive monitoring; each item can be linked to one of the domains [37]. The instrument has been translated to Swedish and validated in a Swedish healthcare context, S-NoMAD [38].

The survey was divided into three parts where part A collected data on the respondents’ characteristics. Part B consisted of general items about PAP, answered on 11-point numeric scales anchored by ‘not at all’ and ‘completely’. Part C consisted of detailed items about PAP, and were answered on 5-point Likert-type scales ranging from ‘strongly disagree’ to ‘strongly agree’. Three additional response options were available if the item was not relevant to the respondents’ role, to PAP, or at this stage of implementation.

The survey was in Swedish, created in Google Forms and was entirely web-based. GPs who participated in the study received a web-link to the survey by email together with participant information in Swedish. Informed consent was given by ticking a box at the beginning of the survey. Participation was anonymous. The survey was open for a total of 33 days and two reminder emails six days apart were sent out to 413 of the potential participants during this time.

### Data analysis

A power calculation was made in R, using the power calculation (pwr) package for multiple linear regression analysis with an assumption of three covariates in the model. With 10% explained variation, 80% power and an alpha level set to 0.05, 102 respondents were required to detect statistically significant associations.

Descriptive statistics were used to analyse response distribution for part A, the general items about PAP in part B and the detailed items in part C (presented in the four NPT domains coherence, cognitive participation, collective action, and reflexive monitoring). The five response categories were merged into three categories as follows: The two categories ‘strongly agree’ and ‘agree’ were merged into ‘agree’, ‘neither agree nor disagree’ was renamed to ‘neutral’, and the two responses ‘disagree’ and ‘strongly disagree’ were merged into ‘disagree’. The additional three response categories, including ‘not relevant to my role’, ‘not relevant at this stage’ and ‘not relevant to PAP’, were excluded from the analysis due to few responses in each category.

Group analyses were made regarding associations to gender, practice location, and years of practice. Gender was dichotomised to man or woman, practice location to Gothenburg or other areas in Region Västra Götaland, and years of practice to over or under 10 years since receiving medical license. The one respondent who did not want to disclose their gender was excluded from the group analyses.

Multiple logistic regression was used to explore associations between GPs likelihood of frequent use of PAP and their gender, practice location, and years of practice. The answers on the item ‘how often do you use PAP?’ was dichotomised to frequently (‘every month’ or ‘every week’) and non-frequently (‘rarely’ or ‘do not use’).

To explore differences between groups regarding the general items in part B answered on 11-point numerical scales, the Mann-Whitney U test was used. For the detailed items in part C, the 5-point Likert-type scales were kept. Responses to the items in part C were summarised in SPSS to one composite score for each domain.

Multiple linear regression was then used to analyse whether the GPs’ gender, practice location, or years of practice significantly predicted their coherence, cognitive participation, collective action, and reflexive monitoring. Significance level was set to 0.05 for all analyses. Internal missing values ranged from 0 to 14 per item after excluding responses as described above. All statistical analyses were performed in IBM SPSS Statistics version 25.

### Ethical considerations

Participation in the study was voluntary. Potential participants received written information about the study and provided informed consent by checking a box in the beginning of the survey. Data were managed and stored so that only the researchers could access them. As no sensitive personal data were collected, the study did not fall under the scope of the Swedish Ethical Review Act and ethical approval by the Swedish Ethical Review Agency was not required.

## Results

### Respondents

Of the 463 GPs invited to participate, 143 completed the survey, corresponding to a response rate of 31%. The response rates were similar for men and women and the majority were GPs.

Over one third of the respondents were employed in Gothenburg and approximately half had more than 10 years of practice (Table 2).

**Table 2.** Respondents’ characteristics.

	n	%
*Gender*		
Women	68	47.6
Men	74	51.7
Does not want to disclose	1	0.7
*Professional category*		
GP	91	63.6
Resident GP	43	30.1
Other licenced physician	9	6.3
*Practice location*		
Fyrbodal	30	21.0
Gothenburg	54	37.8
Skaraborg	10	7.0
Södra Bohuslän	34	23.8
Södra Älvsborg	15	10.5
*Years of practice*		
<1	10	7.0
1-2	16	11.2
3-5	24	16.8
6-10	24	16.8
11-15	25	17.5
>15	44	30.8
*Private or public healthcare centre*		
Private	78	54.5
Public	65	45.5

GP: General practitioner.

### Use of PAP

About one quarter (27%) of the surveyed GPs reported using PAP every week or every month while over half of the respondents (54%) reported using the method less frequently. Nineteen percent stated they do not use PAP. Most (78%) felt that PAP was familiar. Of those who reported using PAP; 83% were familiar with the method, compared to 48% who were familiar with but did not use PAP (*χ*^2^_(1)_ = 13.087; *p <* .001). More respondents (69%) reported that other healthcare professionals at the HCCs use PAP rather than physicians (58%). Thirty-nine percent reported feeling that PAP was a normal part of their work, while (78%) believed it would be in the future.

Significant associations between frequent use of PAP and both practice location and years of practice were seen, while there was no association to gender (Table 3). The strongest predictor was practice location, with GPs in Gothenburg being more than 6 times more likely to prescribe PAP every month or more often than GPs in other areas in the region (OR = 6.4; 95% CI 2.7–14.8). GPs with more than 10 years of practice were more likely to prescribe PAP every month or more often than less experienced GPs (OR = 2.5; 95% CI 1.1–6.0). The model explained 22% of the variance in the reported use of PAP.

**Table 3.** Associations between likelihood of frequent use of Physical activity on prescription and gender, practice location, and years of practice.

	B	SE	*p*	OR	95% CI for OR
Gender^1^	0.298	0.416	0.474	1.347	0.596 to 3.047
Practice location^2^	1.849	0.433	**<0.001**	6.352	2.720 to 14.834
Years of practice^3^	0.935	0.433	**0.031**	2.547	1.090 to 5.955

1 = Groups defined as ‘woman (ref)’ or ‘man’.

2 = Groups defined as ‘Gothenburg’ (ref.) or ‘other areas in Region Västra Götaland’.

3 = Groups defined as ‘<10 years since receiving medical license’ (ref.) or ‘>10 years since receiving medical licence’.

Analysis: Logistic regression.

R^2^_Nagelkerke_: 0.22.

B: Beta coefficient; OR: Odds Ratio: CI: confidence interval.

Bold numbers indicate significant p-values.

Female GPs were more positive than their male colleagues to whether PAP will become a natural part of their work (median = 8 vs 6, *z*= −2.93, *p*=.003). GPs in Gothenburg felt to a greater extent than GPs in other areas that PAP is currently a natural part of their work (median = 5 vs 3, *z*= −3.298, *p*<.001), but there was no significant difference in whether they felt PAP could become a natural part of their work. GPs with more than 10 years of experience were more familiar with using PAP than less experienced GPs (median = 6 vs 8, *z*= −4.128, *p*<.001), and they also felt to a greater extent that PAP is currently a natural part of their work (median = 3 vs 4.5, *z*= −2.644, *p*=.008).

### Coherence

From the four items corresponding to coherence in the NPT, most respondents (70%) agreed that they understand how PAP differs from usual ways of working with PA. Most (61%) stated that they can see the potential value of PAP for their work while almost half (47%) was neutral to whether the staff in their workplace have a shared understanding of the purpose of PAP (Table 4).

**Table 4.** Responses for all items in part C of the survey, presented in their respective Normalisation Process Theory domain.

	Agree *n (%)*	Neutral *n (%)*	Disagree *n (%)*	Excluded or missing *n*
*Coherence*				
I can see how PAP differs from usual ways of working	97 (70)	40 (29)	2 (1)	4
Staff in this organisation have a shared understanding of the purpose of PAP	60 (44)	65 (47)	12 (9)	6
I understand how PAP affects the nature of my work	54 (42)	61 (47)	15 (12)	13
I can see the potential value of PAP for my work	82 (61)	40 (30)	13 (10)	8
*Cognitive participation*				
There are key people who drive PAP forward and get others involved	52 (39)	29 (22)	51 (39)	11
I believe that participating in PAP is a legitimate part of my role	65 (47)	53 (39)	19 (14)	6
I’m open to working with colleagues in new ways to use PAP	115 (82)	20 (14)	6 (4)	2
I will continue to support PAP	96 (70)	37 (27)	5 (4)	5
*Collective action*				
I can easily integrate PAP into my existing work	65 (47)	44 (32)	29 (21)	5
PAP disrupts working relationships	9 (7)	33 (24)	95 (69)	6
I have confidence in other people’s ability to use PAP	91 (65)	41 (29)	9 (6)	2
Work is assigned to those with skills appropriate to PAP	69 (49)	59 (42)	12 (9)	3
Sufficient training is provided to enable staff to implement PAP	30 (21)	52 (37)	60 (42)	1
Sufficient resources are available to support PAP	39 (27)	58 (41)	45 (32)	1
Management adequately supports PAP	57 (41)	68 (49)	15 (11)	3
*Reflexive monitoring*				
I am aware of reports about the effects of PAP	65 (46)	42 (30)	33 (24)	3
The staff agree that PAP is worthwhile	47 (34)	69 (50)	23 (17)	4
I value the effects that PAP has on my work	43 (32)	68 (50)	25 (18)	7
Feedback about PAP can be used to improve it in the future	93 (68)	42 (31)	2 (1)	6
I can modify how I work with PAP	87 (64)	44 (32)	5 (4)	7

PAP: Physical activity on prescription.

The multiple linear regression analysis showed that practice location significantly predicted coherence when controlled for gender and years of practice; GPs in Gothenburg scored higher in the coherence domain than GPs in other areas after controlling for the other two variables (Table 5). The model explained 6% of the variance.

**Table 5.** Associations between dichotomised groups’ composite scores for each Normalization Process Theory domain.

	B	95% CI	*p*	Adj. R^2^
*Coherence*				0.057
Gender^1^	−0.086	−0.427 to 0.256	0.621	
Practice location^2^	−0.484	−0.836 to −0.133	**0.007**	
Years of practice^3^	0.319	−0.024 to 0.633	0.068	
*Cognitive participation*				0.126
Gender^1^	−0.402	−0.735 to −0.069	**0.018**	
Practice location^2^	−0.624	−0.967 to −0.281	**<0.001**	
Years of practice^3^	0.241	−0.094 to 0.576	0.157	
*Collective action*				0.157
Gender^1^	−0.056	−0.374 to 0.261	0.726	
Practice location^2^	−0.738	−1.065 to −0.411	**<0.001**	
Years of practice^3^	0.474	0.154 to 0.793	**0.004**	
*Reflexive monitoring*				0.130
Gender^1^	−0.241	−0.572 to 0.091	0.153	
Practice location^2^	−0.498	−0.839 to −0.156	**0.005**	
Years of practice^3^	0.561	0.227 to 0.895	**0.001**	

^1^Groups defined as ‘woman’ (ref.) or ‘man’.

^2^Groups defined as ‘Gothenburg’ (ref.) or ‘other areas in Region Västra Götaland’.

^3^Groups defined as ‘<10 years since receiving medical license’ (ref.) or ‘>10 years since receiving medical licence’.

Analysis: Multiple linear regression.

B: Beta coefficient; CI: Confidence interval; Adj. R^2^: Adjusted determination coefficient.

Bold numbers indicate significant p-values.

### Cognitive participation

From the four items corresponding to cognitive participation, equal proportions of the respondents agreed and disagreed (39% each) that there are key people who drive PAP forward and get others involved. Almost half (47%) believed that using PAP was a legitimate part of their role while 14% disagreed. Most respondents (82%) reported being open to working with colleagues in new ways to use PAP and 70% responded that they will continue to support PAP (Table 4).

Both GPs’ gender and practice location significantly predicted cognitive participation when controlled for the other variable and years of practice; female GPs scored higher in the cognitive participation domain than their male colleagues and GPs in Gothenburg scored higher in the cognitive participation domain than those in other areas (Table 5). The model explained 13% of the variance.

### Collective action

From the seven items corresponding to collective action the analysis showed that 47% agreed while 21% disagreed that they can easily integrate PAP into their existing work. Many (65%) responded that they have confidence in other people’s ability to use PAP and very few (9%) disagreed to PAP being assigned to those with appropriate skills. On the other hand, 42% disagreed that sufficient training is provided to enable staff to implement PAP while 21% agreed. More responders disagreed (32%) than agreed (27%) that sufficient resources are available to support PAP. Almost half (49%) were neutral to whether management adequately supports PAP while 41% agreed and 11% disagreed (Table 4).

Both practice location and years of practice significantly predicted collective action when controlled for the other variable and gender. GPs in Gothenburg scored higher in the collective action domain than their colleagues in other areas and GPs with more than 10 years of practice scored higher in the ­collective action domain than those with less experience (Table 5). This model explained 16% of the variance.

### Reflexive monitoring

From the five items corresponding to reflexive monitoring, less than half (46%) agreed to being aware of reports about the effect of PAP. There were ambiguous results regarding whether the respondents thought the staff agree that PAP is worthwhile; 50% were neutral, 34% agreed and 17% disagreed. Similar results were seen regarding whether the respondents value the effect PAP has on their work; 50% were neutral, 32% agreed and 18% disagreed. Most (64%) also agreed that they can modify how they work with PAP (Table 4).

Both practice location and years of practice significantly predicted reflexive monitoring when controlled for the other variable and gender. GPs in Gothenburg scored higher in reflexive monitoring than GPs in other areas, and GPs with more than 10 years of practice scored higher in reflexive monitoring than those with less experience (Table 5). This model explained 13% of the variance.

## Discussion

This cross-sectional survey showed that the views on PAP were generally positive amongst GPs in Region Västra Götaland, but only 27% reported using the method regularly. The most prominent barriers for implementing PAP were insufficient training and insufficient resources available, pertaining to the domain *collective action* (The actual work done by GPs in an HCC to implement PAP). Other barriers identified included a lack of key people who drive PAP forward, lack of knowledge about the effects of PAP, and sometimes limited management support. The most important facilitators were positive views on PAP as a method, an understanding of the benefits and value of PAP (*coherence*) and willingness to collaborate with colleagues in using PAP (*cognitive participation*). Most of the respondents felt that PAP could become a natural part of their work. Most respondents considered PAP to be a relevant part of their role as GPs and stated they will continue to support PAP.

Practice location was associated with almost all investigated variables. General practitioners in Gothenburg used PAP to a greater extent and had a more positive view on PAP regarding all four domains in the NPT than GPs in other areas of the region. General practitioners with more than 10 years of practice used PAP to a greater extent and had a more positive view on PAP regarding *collective action* (the operational work to implement PAP) and *reflexive monitoring* (assessing the effects of PAP on themselves and patients) than less experienced GPs. Gender was only associated with one of the investigated domains; *cognitive participation* (the relational work that builds a common PAP practice).

The generally positive scores on the domains *coherence* and *cognitive participation* indicate that the GPs understand the benefits and purpose of using PAP and that they are positive towards working with and developing PAP as a method together with other colleagues. Three of the five biggest perceived barriers were found in the domain *collective action,* indicating this is where the implementation work needs to improve the most. Going from appreciating and understanding the method to use it in clinical practice seems to be a big barrier and working with the items in this domain could increase the use of PAP.

The identified barriers and facilitators are similar to those presented in a recent national report from the Swedish Public Health Agency: lack of time, resources and knowledge hinders the work with PAP in Sweden’s healthcare regions [34]. Previous qualitative studies also consistently reported insufficient training as a barrier for implementing the method [32, 33]. From the patient perspective, chronic pain has been described as an important barrier for increasing PA level [39].

International studies have reported similar barriers also for other PA promotion methods; studies from other European countries and Canada show that insufficient knowledge about PA promoting methods and concerns regarding skills in promoting PA are major barriers amongst professionals in primary care [40–43]. Implementation of Swedish PAP seems to have come further than that of the German equivalent; a study showed that 74% of participating German physicians had never even heard of PAP and less than two percent used the method monthly or more often [40], compared to the 27% in our study. A Canadian study showed that a one-day workshop about the Canadian equivalent to PAP resulted in increased knowledge, confidence in using the method, increased use, and lowered perception of barriers at 3 months after the workshop [41]. Increased education and training in using Swedish PAP may therefore facilitate implementation of the method in areas where it is not yet embedded in everyday clinical practice. With increased education and training in using PAP it is possible that the other perceived barriers, such as support from management and lack of key people who drive PAP forward, will be reduced, which could further facilitate implementation.

A recent survey in Swedish paediatric healthcare, also in Region Västra Götaland and also based on the NoMAD instrument, showed that Swedish PAP was familiar and perceived as an acceptable, appropriate, and feasible intervention, and by many of the participating healthcare practitioners viewed as a normal part of clinical routines, more so by physiotherapists and nurses than by physicians [44]. As in our study, barriers and facilitators were mainly identified in the domains collective action and reflexive monitoring, with the main barrier being a lack of training and of resources to work with PAP to a greater extent. While education and training in the PAP method nowadays is included in undergraduate education for most healthcare professions, including physicians, it seems that training should be provided to practitioners who completed medical school or other undergraduate programmes before it was part of the curriculum.

Our study adds new knowledge about the associations between use and views on PAP and gender, practice location and years of practice. The distinct difference between GPs in Gothenburg and GPs in other areas in the region, both regarding use and views on PAP, is most likely the result of the more extensive implementation work and support structures available in Gothenburg [35]. The findings indicate that the implementation work has a positive effect and by establishing a PAP supporting organisation throughout the region the use of PAP in other areas could be facilitated and increased and would contribute to more equal health care. The study also showed that more experienced GPs used PAP to a greater extent than less experienced GPs. By introducing and increasing education about PAP and training in using the method early in a GP’s work life, less experienced GPs may become more comfortable with working with PAP. Even if the use of PAP is lower amongst GPs in other areas in the region and in GPs with less than 10 years of practice, these groups were still positive that PAP could become a natural part of their work.

For future research it would be relevant to build on the findings from our study with a more extensive qualitative study about barriers and facilitators for working with PAP, and subsequently developing and evaluating a strategy for a more structured implementation of PAP in primary healthcare. A comparison between regions would also be interesting to see how well other regions have succeeded in implementing PAP and use this knowledge to develop a national implementation plan. When the implementation work has come further in Region Västra Götaland, a follow-up study could be performed using the same survey as in this study to evaluate the potential improvements in use and views of PAP amongst Swedish GPs.

### Strengths and limitations

The survey response rate of 31% is slightly below that of other recent survey studies about PA promotion amongst physicians [40, 42]. This, together with the fact that only 69 of the 184 invited HCCs agreed to participate, limits the conclusions we can draw as well as the generalisability of the findings. However, distribution of gender, years of practice, and practice location throughout the region was quite even, making the results more representative for the population. Dropout analyses of differences between characteristics of HCCs who participated and those who did not, in terms of publicly funded versus privately owned HCCs, as well as geographic location and disparities between urban and rural areas, showed no significant difference between HCCs that chose to participate and those who did not. The number of respondents also well exceeds the power calculation, which increases the confidence in our findings. Another limitation is that the present study only focused on GPs in one region of Sweden, limiting generalisability to other regions. Therefore, further research in a larger sample that includes other regions is required to fully investigate and follow the implementation of PAP amongst Swedish GPs.

The GPs’ willingness to participate may have introduced a selection bias. Healthcare centre managers at HCCs with more developed routines for PAP may have had a greater interest in participating in the study. The survey may also have been biased towards GPs with stronger opinions and perceptions on PAP, positive or negative.

Major strengths of the study are the use of an implementation theory as well as a validated questionnaire, which increases the study’s internal validity. The questionnaire was already translated and validated in a Swedish healthcare context [38], so no translation was necessary. The use of an implementation theory made the analysis structured and made it possible to better understand and explain what areas need to improve to facilitate implementation. The study yielded several interesting and significant findings and even if the transition to composite scores can make the results more difficult to interpret, it enabled us to analyse the result in relation to the domains in the NPT.

## Conclusions and implications

The study findings suggest that the positive views held by the participating GPs in Region Västra Götaland towards working with PAP are helpful, but that more education and training in using the method need to increase for the method to be implemented successfully in Swedish health care. An increase in resources, management support, and dedicated people who drive PAP forward would also facilitate the implementation. General practitioners in Gothenburg, where the most extensive implementation is done, use PAP more frequently and have a more positive view of PAP. The method is also used more frequently amongst GPs with more than 10 years of practice.

## Data Availability

The data that support the findings of this study are available from the corresponding author, SB, upon reasonable request.

## Note

1 Physical Activity in the Prevention and Treatment of Disease, published by Professional Associations for Physical Activity (YFA)

## List of abbreviations

GP: general practitioner
HCC: healthcare centre
NPT: normalization process theory
PAP: physical activity on prescription
PA: physical activity
WHO: World Health Organization
